# Identification and Functional Analysis of Glutathione S-Transferases from *Sitophilus zeamais* in Olfactory Organ

**DOI:** 10.3390/insects13030259

**Published:** 2022-03-05

**Authors:** Daosong Xia, Renwen Zheng, Jianhua Huang, Sihan Lu, Qingfeng Tang

**Affiliations:** 1 Anhui Province Key Laboratory of Integrated Pest Management on Crops, Key Laboratory of Biology and Sustainable Management of Plant Diseases and Pests of Anhui Higher Education Institutes, College of Plant Protection, Anhui Agricultural University, Hefei 230036, China; xiadaosong_25@163.com (D.X.); zhengrenw@163.com (R.Z.); lusihan0911@163.com (S.L.); 2 Institute of Insect Sciences, College of Agriculture and Biotechnology, Zhejiang University, Hangzhou 310058, China; jhhuang@zju.edu.cn

**Keywords:** *Sitophilus zeamais*, odorant-degrading enzymes, glutathione S-transferases, degradation metabolism

## Abstract

**Simple Summary:**

*Sitophilus zeamais* is a worldwide pest that destroys many grain products, causing a loss of cereal quality and quantity resulting from its metabolites and behavior. Glutathione S-transferases (GSTs), as a group of odorant-degrading enzymes (ODEs), play an important role in degrading xenobiotic odorant molecules in insect olfactory sensing systems. However, there have been few reports about the function of the GST genes of *S. zeamais* in the odorant-degrading process. In this study, we characterized 13 full-length genes encoding GST sequences from *S. zeamais* and analyzed the expression pattern in different tissues of SzeaGSTd1. In addition, we investigated the ability of recombinant SzeaGSTd1 to degrade the volatile molecules of the host, and the data indicated that the content of capryl alcohol significantly decreased in the system. In summary, we believe SzeaGSTd1 plays a key role in the olfactory sensing system of *S. zeamais*.

**Abstract:**

Odorant-degrading enzymes (ODEs) play an important role in rapidly degrading and inactivating odorant molecules that have completed information transmission, as well as in maintaining the stability and sensitivity of insect olfactory sensing systems. Glutathione S-transferases (GSTs), as a group of ODEs, supposedly bear the ability to catalyze the conjugation of glutathione (GSH) and xenobiotic odorant molecules in the degrading process. However, there are few reports regarding the role of the GST genes of *Sitophilus zeamais* in the degrading process. Thus, we characterized 13 full-length genes encoding GST sequences from *S. zeamais*, of which only *SzeaGSTd1* contained a high abundance in the antennae. Ligand-binding assays implied that SzeaGSTd1 was able to catalyze the conjugation of GSH with 2, 4-dinitrochlorobenzene (CDNB). We investigated whether recombinant SzeaGSTd1 bears the ability to degrade the volatile molecules of the host; among the host volatiles, and found capryl alcohol to be a suitable substrate for SzeaGSTd1. These results strongly suggest that SzeaGSTd1 probably plays a role in auxiliary host location by degrading the host volatiles of capryl alcohol and exhibits a potential biological function in the olfactory sensing system of *S. zeamais*. Knowledge of the potential functions of SzeaGSTd1 will provide new ideas for biological control strategies for *S. zeamais*.

## 1. Introduction

*Sitophilus zeamais* is a worldwide pest of cereals, decreasing the quality and quantity of grain products [1,2,3]. The technologies used to control this insect include the use of synthetic pesticides such as phosphine [4,5], but it is now necessary to develop non-chemical control strategies for *S. zeamais* due to the global demand for decreasing pesticide residues in food and resistance to pesticides. An efficient olfactory sensing system plays a vital role in various insect behaviors, helping insects to locate hosts and mate and enabling smooth communication with companions [6,7,8]. Thus, intensive research on olfaction might help us to understand the behavioral responses of insects and identify biological targets for new control strategies in *S. zeamais*.

In the sensilla of insect antennae, odorant-binding proteins (OBPs) or chemosensory proteins (CSPs) can bind and transport volatile semiochemicals to odorant receptors (ORs) [9,10,11]. Then, the information imparted by volatile semiochemicals is recognized and insects respond with physiological behavior accordingly [12,13,14]. After the completion of odorant recognition and information transmission, odorants need to be degraded by a variety of odorant-degrading enzymes (ODE) to terminate the stimulation, ensuring that insects can prepare for the next odorant stimulation and respond to changes in signals [6,15,16]. Based on their substrate specificities, ODEs are divided into different groups, including glutathione S-transferases (GSTs), carboxylesterases (CXEs), cytochrome P450 monooxygenases (P450s), aldehyde oxidases (AOXs), and UDP-glucuronosyltransferases (UGTs) [6,17,18].

In insects, GSTs are found in two categories: cytosolic and microsomal GSTs [19,20]. Cytosolic GSTs are divided into six classes, Delta, Epsilon, Omega, Sigma, Theta, and Zeta, of which the Delta- and Epsilon-class GSTs are insect-specific [21,22]. The high expression of GSTs in insect antennae suggests that they might be related to olfactory function in odorant degradation. Antennal-specific GST (GST-msolf1) was discovered to be located in the sex-pheromone-sensitive sensilla of *Manduca sexta* antennae and could modify aldehyde odorants (trans-2-hexenal), suggesting that the GST-msolf1 might be involved in olfactory system protection through inactivation of aldehyde odorants [23]. GST-pxcs1 is abundantly expressed in the chemosensory organs of *Papilio xuthus*, and it can inferred that GST-pxcs1 plays a role in degrading chemical ligands from the external environment [24]. Meanwhile, in some Lepidopteran species, such as *Bombyx mori* and *Chilo suppressalis*, GST genes were identified and listed as candidates for ODEs, potentially being involved in degradation of volatile odorants in the sensilla [25,26,27]. As the model insect for Coleoptera, *Tribolium castaneuem*’s GSTs have already been annotated and reported [22], and TcasGSTd2 was inferred to be an olfactory-specific GST [28]. However, the functions of GSTs in degrading odorant molecules in *S. zeamais* have not yet been reported. Our study results will contribute to the knowledge on the function of GSTs in the olfactory sensing of *S. zeamais* and help us to develop new biological control strategies.

In this research, we identified 13 cytosolic GST genes from *S. zeamais* antennal transcriptome data. We focused our study on one Delta-class GST: SzeaGSTd1. The expression patterns in different tissues were tested, and purified recombinant SzeaGSTd1 was obtained. We investigated the enzymatic properties of recombinant SzeaGSTd1 and tested the ability of SzeaGSTd1 to interact with odorants using host volatile molecules. These results suggest that SzeaGSTd1 could degrade volatile alcohol odorants (capryl alcohol) and might function as an ODE in *S. zeamais*.

## 2. Materials and Methods

### 2.1. Insects Reared, RNA Extraction and cDNA Synthesis

*Sitophilus zeamais* from the College of Plant Protection, Anhui Agricultural University, were reared on wheat grain under total darkness in glass bottles (26 ± 1 °C and 80 ± 5% relative humidity). To acquire the testing tissues, we dissected the insects into individual tissues (male antennae, female antennae, heads (without antennae), thoraces (without legs and wings), abdomens, legs, and wings).

Total RNA was isolated from cryopreserved tissues by RNAiso Plus (TaKaRa, Dalian, China) based on the manufacturer’s instructions. RNA purity and concentration were tested using the NanoDrop 2000 spectrophotometer (Thermo Fisher Scientific, Waltham, MA, USA). The cDNA was synthesized from previous total RNA by a PrimeScriptTM RT reagent kit with gDNA Eraser (TaKaRa, Dalian, China) on PCR equipment (Bio-Rad S1000, Bio-Rad Laboratories, Hercules, CA, USA), with the PCR conditions based on the reagent instructions. Then, we stored the PCR products at −20 °C until the following experiments took place.

### 2.2. SzeaGST Genes Identification and Sequences Analysis

All SzeaGST sequences were obtained from our previous antennal transcriptome data on *S. zeamais* [29]; the sequence read archive accession number from the National Center for Biotechnology Information (NCBI) was SRX3427302. The SzeaGST sequences were identified using the BLASTX tool (https://blast.ncbi.nlm.nih.gov/Blast.cgi accessed on 22 April 2020) (E-value < 1 × 10^−5^) at NCBI, and the sequence was then manually checked to ensure that it was a valid query [30,31].

The open reading frames (ORFs) were predicted with ORF Finder (www.ncbi.nlm.nih.gov/orffinder accessed on 22 April 2020). The theoretical isoelectric points and molecular weights of the SzeaGSTs were predicted using the ExPASy server (www.expasy.org/tools/protparam.html accessed on 22 April 2020). The phylogenetic tree with a p-distance model and the pairwise deletion of gaps [32] was constructed using MEGA 7.0, while the bootstrap support of tree branches was assessed by resampling the amino acid positions 1000 times.

### 2.3. Bioinformatics Analysis of SzeaGSTd1

The amino acid sequence alignments were produced by DNAMAN 7.0 with the default gap penalty parameters of 10 gap openings and an extension of 0.2 and then edited by GENEDOC 3.2. The secondary structure of the amino acid sequence was predicted by PSIPRED 4.0 (http://bioinf.cs.ucl.ac.uk/psipred/ accessed on 22 April 2020). The web tool SWISSMODEL (https://swissmodel.expasy.org/ accessed on 22 April 2020) was used to predict the 3D structure. The catalytic residues of amino acids were predicted by the CD search tool (https://www.ncbi.nlm.nih.gov/Structure/bwrpsb/bwrpsb.cgi accessed on 22 April 2020).

### 2.4. qRT-PCR

On the Bio-Rad CFX96 Real-Time PCR System, cDNA samples of *SzeaGSTs* were tested through qRT-PCR, and endogenous reference selected the *β*-actin gene of *S. zeamais*. The primers (list in Table S1) were designed by Primer Premier 5. The qRT-PCR cycling parameters were set according to the manufacturer’s instructions and we then measured the melting curves. As a negative control, we used sterilized H_2_O in the reaction mixture instead of sample cDNA. All reactions were performed in three technical replicates and three biological replicates.

The comparative 2^−ΔΔCt^ method [33] was used to quantify the relative expression levels of SzeaGSTd1 in different tissues. Comparative expression analyses were implemented with Tukey’s honestly significant difference (HSD) test (*p* < 0.05) and one-way ANOVA. We analyzed the statistical data using the Data Processing System (DPS) software [34].

### 2.5. Expression and Purification of Recombinant SzeaGSTd1 Protein

On the basis of the ORF sequence of SzeaGSTd1 (accession number of GenBank: MW390709), the specific primers (with *Hind III* and *BamH I* restriction enzyme sites) were designed (Table S1) for the ORF of SzeaGSTd1. The following conditions were employed for the PCR reaction with the TaKaRa Ex Taq^®^ DNA Polymerase (TaKaRa, Dalian, China): 98 °C for 30 s, followed by 35 cycles at 98 °C for 10 s, 55 °C for 30 s, and 72 °C for 1 min, with a final extension at 72 °C for 2 min. Here, 50 μL of reaction mixture consisted of: 0.25 μL of Ex Taq, 5 μL of 10 × Ex Taq buffer, 4 μL of dNTP mixture, 2 μL of cDNA, 2 μL of each primer (10 μM), and 34.75 μL of sterilized H_2_O. The products were inserted into pMD 19-T Vector (TaKaRa, Dalian, China) and sequenced by General Biosystems (Hefei, China).

The plasmid with the ORF of SzeaGSTd1 was digested with HamH I and Hind III and ligated into the expression vector pCold I. Then, the correct resulting pCold I-SzeaGSTd1 was transformed into *Escherichia coli* BL21 cells (TaKaRa, Dalian, China) for expression. Bacteria were cultured in a Luria–Bertani (LB) medium (containing 50 μg/mL of ampicillin, 20 μg/mL of chloramphenicol, and 5 μg/mL of tetracycline) and grown at 37 °C with shaking at 220 rpm until the OD600 reached 0.4–0.5. Then, a 0.1 mM final concentration of isopropyl β-D-thiogalactopyranoside (IPTG) was added to the medium to induce the expression of recombinant SzeaGSTd1 protein at 15 °C with shaking at 220 rpm for 24 h. The bacterial cells were harvested by centrifugation at 4 °C and 12,000× *g* for 5 min, then resuspended in phosphate-buffered saline (PBS, pH 7.2). At 4 °C, the cells were centrifuged at 12,000× *g* for 20 min after being sonicated for 20 min to separate the supernatant. The recombinant SzeaGSTd1 proteins were in soluble form. Recombinant proteins were purified using Ni-His resin and eluted using an ascending series of imidazole (10–300 mM) in balance buffer. The purified protein was dialyzed using 100 mM of sodium phosphate buffer (pH 7.2). The recombinant SzeaGSTd1 was analyzed through 12% sodium dodecyl sulfate polyacrylamide gel electrophoresis (SDS-PAGE), and the concentrations were determined using the BCA Protein Assay Kit (TaKaRa, Dalian, China).

### 2.6. Enzyme Activity Assay

The activity of SzeaGSTd1 was measured using 1-chloro-2,4-dinitrobenzene (CDNB) as the standard substrate, following the procedure adapted from Habig et al. [35]. Briefly, 4 μg of recombinant SzeaGSTd1 protein was added to 100 mM of phosphate buffer (pH 7.2, containing a final concentration 1mM of GSH and different concentrations of CDNB) with a total reaction volume of 200 μL. Changes in absorbance at 340 nm (A340) at 1 min intervals for 5 min were monitored using Spectrophotometer 1510 (Thermo Fisher Scientific Oy, Finland). Kinetic parameters were calculated using the Michaelis–Menten plot in GraphPad Prism 5 (San Diego, CA, USA), with the data produced by the assay conditions with different concentrations of CDNB. For measuring the optimum temperature and pH of SzeaGSTd1, the concentrations of GSH and CDNB were fixed at 1 mM and 2 mM, respectively. The optimum pH was determined in 100 mM of PBS buffer (with a pH range of 5.0–9.0), and the optimum temperature was measured after preincubating recombinant SzeaGSTd1 at various temperature ranges (10–60 °C) for 30 min. Based on the competition between the host volatiles and the CDNB, we added various host volatiles to an enzyme activity reaction system to measure the interaction of SzeaGSTd1 and host volatiles. We selected 10 host volatiles (Table S2) that had been reported as food-sourced volatiles of *S. zeamais* [36,37,38,39,40,41,42,43,44,45,46], and the host volatile solutions were prepared in methanol. The host volatiles (final concentration 50 μM) were preincubated with 2 μg of recombinant SzeaGSTd1 for 10 min at 30 °C before being added in a total volume of 200 μL to the reaction system (containing a final concentration of 1mM of GSH and 2 mM CDNB). Recombinant protein without the addition of host volatiles was employed as the control. The assays were conducted in at least three biological and experimental replicates.

### 2.7. Metabolism Assays In Vitro

The capability of recombinant SzeaGSTd1 to metabolize host volatiles (capryl alcohol, vanillin, and benzaldehyde) was analyzed using high-performance liquid chromatography (HPLC) on the Waters 600E equipment (Waters Corporation, Milford, MA, USA). For this purpose, the total 500 μL volume reaction system containing 100 mM of PBS (pH 7.2), 2.5 mM of GSH, 20 μg of recombinant SzeaGSTd1, and 1 μg/mL of vanillin or 1 mM capryl alcohol or benzaldehyde was incubated at 30 °C with shaking at 200 rpm for 0.5 h; then, 500 μL methanol (HPLC grade) was added to stop the reaction. Subsequently, the reaction mixture was centrifuged at 12,000× *g* for 20 min, and 500 μL of supernatant was filtered into HPLC vials via a 0.22 mm organic membrane. When the sample (20 μL) was injected into a C18 column (4.6 × 250 mm, 5 µm, Waters Corporation, Milford, CT, USA), the residual content of host volatiles was analyzed with the mobile phase of 80% acetonitrile and 20% water with a 1 mL/min flow rate at 30 °C. The absorbance wavelength for capryl alcohol was set to 206 nm, that for vanillin was set to 230 nm, and that for benzaldehyde set to 237 nm. A heat-inactivated enzyme was used as the control. The experiments were performed with three replicates.

## 3. Results

### 3.1. Identification and Analysis of SzeaGSTs

**Figure 1:**
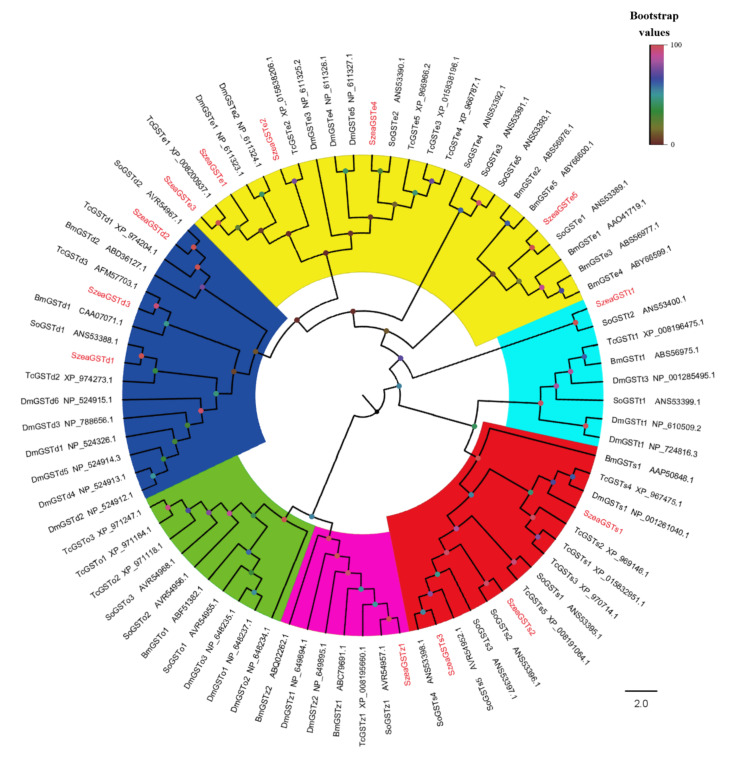
Phylogenetic analysis of SzeaGSTs with GSTs of other insects. The GST family is shown in different colors: blue: Delta; red: Sigma; yellow: Epsilon; green: Omega; sky blue: Theta; purple: Zeta; So: *Sitophilus oryzae*; Tc: *Tribolium castaneum*; Dm: *Drosophila melanogaster*; Bm: *Bombyx mori*.

We identified 13 SzeaGST genes from the antennal transcriptome data and named them *SzeaGSTd1*~*SzeaGSTt1.* The sequence of *SzeaGSTs* was submitted to GenBank (MW390709-MW390721), and the predicted molecular mass (MW) and theoretical pI of the protein are shown in Table 1. All *SzeaGST* gene sequences comprised 606 to 744 bp (all included complete ORF), encoded 116 to 239 amino acid residues, and exhibited a high identity with those of other Coleoptera insects (83.56% to 99.17% identity match with *Sitophilus oryzae* and *Lissorhoptrus oryzophilus*) based on the result of BLASTX best hit (Table 1). For the phylogenetic analysis, 68 amino acid sequences of known insect GST genes were selected to build a phylogenetic tree with the sequences of SzeaGSTs (Figure 1), and all SzeaGSTs were clustered together into already known insect GST clades (Delta, Epsilon, Sigma, Theta, and Zeta classes).

**Figure 2:**
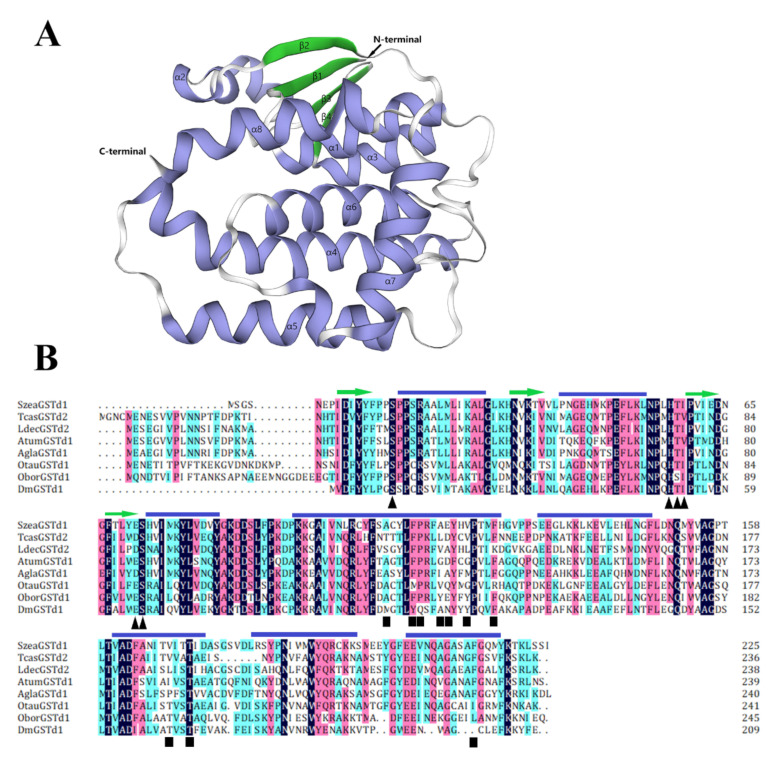
Amino acid sequence structure analysis of SzeaGSTd1. (**A**) The modeled 3D structure of SzeaGSTd1 (DmGSTd1 (Protein Data Bank ID: 3mak [47]) of *D. melanogaster* is the structural template). The α-helices and β-sheets were labeled with α and β, respectively. (**B**) Sequence alignment of delta-class GSTs from insects. Black triangles indicate amino acid residues that comprise the G site and black squares indicate the amino acid residues that comprise the H site. The position of β-sheets (β1-β4, indicated by the green arrow) and α-helices (α1-α8, indicated by the blue line segment) in the SzeaGSTd1 protein sequence is shown on top of the alignment. TcasGSTd2 from *T. castaneum*, QES86455.1; LdecGSTd2 from *Leptinotarsa decemlineata*, XP_023021411.1; AtumGSTd1 from *Aethina tumida*, XP_019870085.1; AglaGSTd1 from *Anoplophora glabripennis*, XP_018560761.1; OtauGSTd1 from *Onthophagus taurus*, XP_022909213.1; OborGSTd1 from *Oryctes borbonicus*, KRT81939.1; DmGSTd2 from *D. melanogaster*, NP_524326.1.

The full-length sequence of *SzeaGSTd1* is 744 bp and encodes the proteins of 226 amino acids. The protein predicted MW is 25.55 and the pI is 6.65 (Table 1). The sequence of SzeaGSTd1 exhibits a high amino acid identity with other known insect GSTs, such as TcasGSTd2 (QES86455.1, *Tribolium castaneum*, 50% identity), LdecGSTd2 (XP_023021411.1, *Leptinotarsa decemlineata*, 48% identity), AtumGSTd1 (XP_019870085.1, *Aethina tumida*, 45% identity), AglaGSTd1 (XP_018560761.1, *Anoplophora glabripennis*, 48% identity), OtauGSTd1 (XP_022909213.1, *Onthophagus taurus*, 43% identity), and OborGSTd1 (KRT81939.1, *Oryctes borbonicus*, 43% identity). According to the results of template matching, DmGSTd1 of *Drosophila melanogaster* (Protein Data Bank ID: 3mak) [47] was selected as the structural template for the SzeaGSTd1 construction model, and the sequence homology between the template and SzeaGSTd1 was 45.67%. The GMQE structural validation (0.75) and QMEAN Z score (−0.59) of the SzeaGSTd1 model showed that the quality of the SzeaGSTd1 model is very high. The structure of SzeaGSTd1 bears the typical structural characteristics of GST fold (Figure 2A); the N-terminal domain has a motif topology (β_1_-α_1_-β_2_-α_2_-β_3_-β_4_-α_3_), and the C-terminal domain contains five helices (α_4_-α_8_), resembling a right-handed α-helical bundle. The results of the SzeaGSTd1 sequence analysis (Figure 2B) revealed that the N-terminal domain of SzeaGSTd1 with the G site is conserved, indicating the presence of the same GSH-binding mechanism. However, the C-terminal domain with the H-site exhibits a low sequence identity, meaning that it might be responsible for the differences and diversities observed in substrate selectivity.

### 3.2. The Spatial Expression of SzeaGSTs

**Figure 3:**
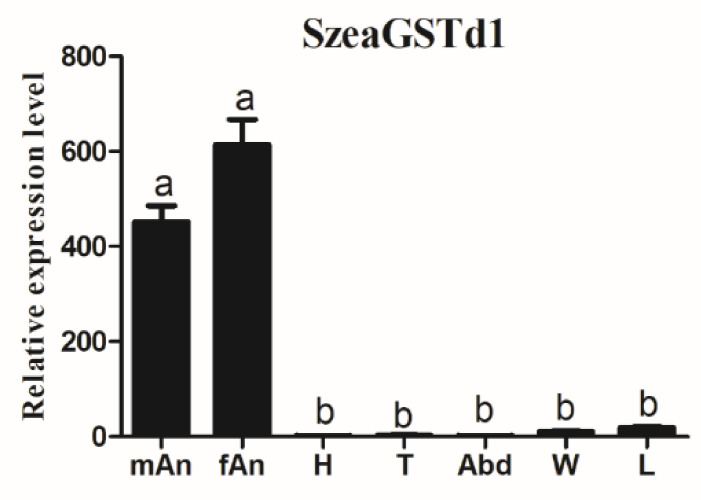
The relative expression levels of *SzeaGSTd1* in different tissues. mAn: male antenna; fAn: female antenna; H: head; T: thorax; Abd: abdomen; W: wing; L: leg. The error bars represent the standard errors calculated from three replicates. Different letters on the error bars indicate significant differences analyzed by the ANOVA and HSD test (*p* < 0.05).

The results of the tissue expression patterns of the *SzeaGSTd1* indicated its expression in all tested tissues, but was more significantly expressed in the antennae than in other non-olfactory tissues (Figure 3). The tissue distribution of other *SzeaGSTs* is shown in Figure S1. *SzeaGSTd2*, *SzeaGSTe1*, *SzeaGSTe4*, and *SzeaGSTs3* were expressed in all tissues, but the expression level measured the highest in male antennae. *SzeaGSTe3* and *SzeaGSTs1* were expressed predominately in the leg. *SzeaGSTe2*, *SzeaGSTe5*, and *SzeaGSTz1* were expressed significantly in the wing. *SzeaGSTd3* was expressed predominately in the antennae, wings, and legs. *SzeaGSTs3* was expressed in all tissues, but abundantly expressed in the abdomen. *SzeaGSTt1* was significantly expressed in the head. According to the results of the tissue-specific expression profiles, we chose to use *SzeaGSTd1* for the functional analysis.

### 3.3. Biochemical Characterization of Recombinant SzeaGSTd1

**Figure 4:**
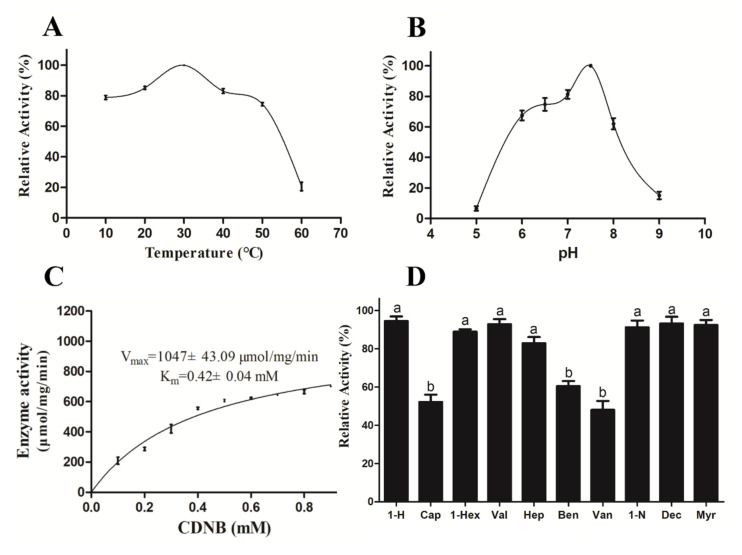
Enzymatic properties of recombinant SzeaGSTd1. (**A**) Relative activity of recombinant SzeaGSTd1 at different temperatures. (**B**) Relative activity of recombinant SzeaGSTd1 at different pH levels. (**C**) The enzyme kinetic properties of recombinant SzeaGSTd1 to CDNB. (**D**) Effect of host plant volatiles on recombinant SzeaGSTd1. 1-H: 1-Hexanol; Cap: capryl alcohol; 1-Hex: 1-Hexadecanol; Val: Valeraldehyde; Hep: Heptaldehyde; Ben: Benzaldehyde; Van: Vanillin; 1-N: 1-Nonanal; Dec: Decanal; Myr: Myrcene. Error bars represent mean ± S.D. of three independent experiments conducted in three replicates. Different letters on the error bars indicate significant differences identified by ANOVA and HSD tests (*p* < 0.05).

In the *E. coli* BL21, the recombinant SzeaGSTd1 was mainly expressed as soluble protein. The expected MW of recombinant SzeaGSTd1 (containing tags of expression vector pCold I) is approximately 28 kDa, and SDS−PAGE results indicated that the SzeaGSTd1 was shown as an estimated 28 kDa single band (Figure S2). At pH 7.5 and 30 °C, recombinant SzeaGSTd1 exhibited optimum catalytic activity (Figure 4A,B). At the GSH concentration of 1 mM, the CDNB-conjugating activities of SzeaGSTd1 in response to different concentrations of CDNB were determined, and the kinetic parameter was analyzed according to the Michaelis–Menten plot (Figure 4C). The V_max_ was 1047 ± 43.09 μmol/mg/min, the K_m_ was 0.42 ± 0.04 mM, the catalytic number K_cat_ was 444.97 s^−1^, and the catalytic efficiency (K_cat_/K_m_) was 1.06 × 10^4^ s^−1^ Mm^−1^.

### 3.4. Substrate Identification and In Vitro Metabolism with Recombinant SzeaGSTd1

We measured the interaction of SzeaGSTd1 and host volatiles molecules using a previously described method (the competition between host volatile molecules and CDNB) of Daniel Gonzalez et al. [48]. We selected ten different host volatiles to measure the rate of inhibition of SzeaGSTd1 activity. The host volatiles were preincubated with SzeaGSTd1 for 10 min before being added to 1 mM of GSH and 2 mM of CDNB; then, the activity test was performed. The results (Figure 4D) indicate that capryl alcohol, benzaldehyde, and vanillin exhibited the strongest inhibitory effects on SzeaGSTd1 activity (47.76%, 39.74%, and 51.94% inhibition, respectively), among the selected host volatile molecules. The inhibition rates of these three host volatiles were significantly higher than others found, and the inhibition effects seen between 1-Hexanol, 1-Hexadecanol, Valeraldehyde, Heptaldehyde, 1-Nonanal, Decanal, and Myrcene were not significantly different. In light of this, the main function of SzeaGSTd1 might be in the metabolism of host volatile molecules.

**Figure 5:**
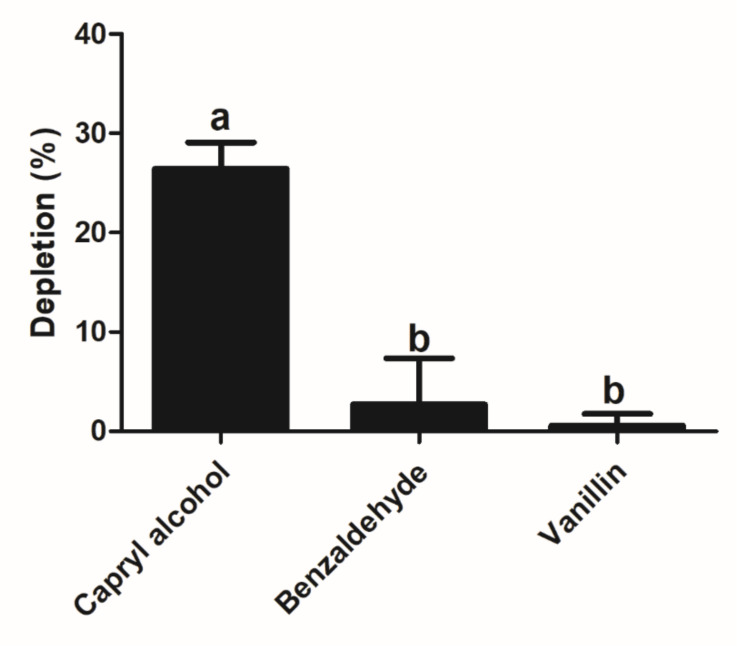
The degradation abilities of recombinant SzeaGSTd1 for capryl alcohol, benzaldehyde, and vanillin. Error bars represent mean ± S.D. of three independent experiments conducted in three replicates. Different letters on the error bars indicate significant differences identified by ANOVA and HSD tests (*p* < 0.05).

Based on the three host volatiles of biological significance, metabolism assays of capryl alcohol, benzaldehyde, and vanillin were carried out in vitro. The results showed that the recombinant SzeaGSTd1 had the ability to degrade capryl alcohol by about 26.45% in 30 min, but the metabolisms of benzaldehyde and vanillin were not significantly changed (Figure 5, Table S3, Figures S3 and S4).

## 4. Discussion

GSTs are vital enzymes in insects which are crucial in the olfactory sensory systems of insect antennae [21,23,49]. Therefore, we identified 13 cytosolic GSTs from the antennal transcriptome datasets of *S. zeamais* (SRX3427302), including three Delta-class (*SzeaGSTd1*-*SzeaGSTd3*), five Epsilon-class (*SzeaGSTe1*-*SzeaGSTe5*), three Sigma-class (*SzeaGSTs1*-*SzeaGSTs3*), and one Theta- and Zeta-class (*SzeaGSTt1* and *SzeaGSTz1*) GSTs. Compared to the Tribolium, the number of identified GST genes represents about one-quarter of the total, only one GST gene is specifically expressed in the antennae, and eight GST genes are significantly enriched in the antennae. The expression of the Tribolium is different in different tissues, and only TcasGSTe18 showed a high expression in the leg [28]. Our identified results are consistent with those of a previous study, in that 9 GSTs were identified in *Aphidius gifuensis* and *Dendroctonus armandi* [50,51], 12 cytosolic GSTs were identified in *Cydia pomonella*, and 16 GSTs were identified in the antennal transcriptome of *Chilo suppressalis* [25,27]. If the GSTs function in odorant degradation as the olfactory genes of insects, they will generally show preferential expression in the antennae. In the antennae of *M. sexta*, GST-msolf1 was expressed specifically and could degrade aldehyde odorants (trans-2-hexenal) [23]. The GST-pxcs1 was preferentially expressed in chemosensory organs of *P. xuthus*, which was inferred to degrade chemical odorants [24]. In *Grapholita molesta*, GmolGSTD1 was highly expressed in the antennae and could degrade (Z)-8-dodecenyl alcohol of the sex pheromone effectively, presuming that it could protect the olfactory system by acting as an ODE [52]. The antennae are an important olfactory organ and crucial in various insect behaviors due to its ability to recognize diverse chemical odorants. Thus, we selected the candidates of SzeaODEs for the expression pattern analysis, and the results showed that SzeaGSTd1 was specifically expressed in the antennae. Our results indicate that SzeaGSTd1 might play a physiological role in the olfactory sensory system of *S. zeamais*, but the key issues remain regarding whether SzeaGSTd1 could degrade odorants and what types of odorant could be degraded.

The principal function of GSTs is to catalyze various compounds with the conjugation of reduced GSH in order to metabolize or sequester the compounds directly. To investigate whether SzeaGSTd1 could degrade odorants, SzeaGSTd1 was expressed in vitro and purified. Then, we assayed the enzyme activity of recombinant SzeaGSTd1 by using CDNB as a substrate, and the results indicated that recombinant SzeaGSTd1 could catalyze CDNB with the conjugation of reduced GSH. When a variety of substrates in the enzyme reaction system are present, the substrate has a competitive relationship with the enzyme, and there will be a competitive inhibition relationship between the substrates, resulting in reduced enzyme activity of the substrate, which is shown by competitive inhibition in enzyme activity. Therefore, by adding odorant compounds to the enzyme activity determination system, we detected their competitive inhibition in the reaction of CDNB and GSH catalyzed by recombinant protein SzeaGSTd1 so as to verify whether the SzeaGSTd1 enzyme protein exhibits a certain binding and degradation effect on odorant compounds. In this paper, the experimental results showed that the SzeaGSTd1 enzyme protein can bind to host volatiles, and its activity to the common substrate CDNB of GST is also inhibited by volatile compounds, which indicates the presence of a competitive inhibition relationship between odorant compounds and CDNB—that is, odorant molecules “compete” for the catalytic ability of SzeaGSTd1 enzyme protein in the substrate in the reaction system. Here, we tested the ability of recombinant SzeaGSTd1 to interact with host volatile molecules. Among the ten volatile molecules, capryl alcohol, benzaldehyde, and vanillin displayed the strongest inhibitory effects on recombinant SzeaGSTd1 activity. Our data indicate that these volatile molecules exhibit a strong binding affinity with SzeaGSTd1 and suggest these as potential substrates. We used HPLC to measure whether the recombinant SzeaGSTd1 could degrade host volatile molecules in vitro, with subsequent results showing that the recombinant SzeaGSTd1 functions to selectively degrade capryl alcohol by about 26.45% in 30 min. Previous studies have shown that capryl alcohol is a volatile component of stored grain and that the content increases significantly during the storage period [53,54]. Therefore, degradation of the residual capryl alcohol in the olfactory system of *S. zeamais* might contribute to locating host food and the optimum oviposition site for females.

## 5. Conclusions

In conclusion, we demonstrate that SzeaGSTd1 might play an important role in olfactory sensory system protection and alcohol odorant inactivation for *S. zeamais*. This promising fundamental knowledge on cereal–insect interactions may pave the way toward the development of novel insect pest management strategies.

## Figures and Tables

**Table 1:** Summary of the GST genes identified in *Sitophuls zeamais*.

Gene Name	Length (bp)	GenBank Acc. No.	Mw (kDa)	pI	BLASTX Best Hit			
					Species	Acc. No.	E-Value	Identity (%)
*SzeaGSTd1*	744	MW390709	25.55	6.65	*Sitophilus oryzae*	XP_030752074.1	1 × 10^−151^	98.23
*SzeaGSTd2*	666	MW390710	24.04	4.95	*Sitophilus oryzae*	XP_030764913.1	3 × 10^−159^	98.64
*SzeaGSTd3*	651	MW390711	24.38	5.30	*Sitophilus oryzae*	XP_030767740.1	1 × 10^−146^	99.17
*SzeaGSTe1*	645	MW390712	22.56	5.44	*Sitophilus oryzae*	XP_030753917.1	6 × 10^−153^	98.13
*SzeaGSTe2*	657	MW390713	23.97	7.89	*Sitophilus oryzae*	XP_030753906.1	1 × 10^−157^	99.08
*SzeaGSTe3*	678	MW390714	25.31	4.75	*Lissorhoptrus oryzophilus*	AVT42199.1	2 × 10^−138^	83.56
*SzeaGSTe4*	654	MW390715	24.50	5.70	*Sitophilus oryzae*	XP_030766193.1	2 × 10^−153^	96.77
*SzeaGSTe5*	639	MW390716	24.23	5.41	*Sitophilus oryzae*	XP_030753847.1	2 × 10^−149^	96.70
*SzeaGSTs1*	606	MW390717	21.88	5.96	*Sitophilus oryzae*	XP_030749148.1	3 × 10^−145^	99.00
*SzeaGSTs2*	612	MW390718	13.40	8.98	*Sitophilus oryzae*	XP_030758386.1	2 × 10^−142^	98.03
*SzeaGSTs3*	609	MW390719	23.26	7.70	*Sitophilus oryzae*	AVR54952.1	2 × 10^−125^	85.64
*SzeaGSTz1*	654	MW390720	18.39	6.37	*Sitophilus oryzae*	AVR54957.1	2 × 10^−^^158^	99.08
*SzeaGSTt1*	720	MW390721	27.80	5.41	*Sitophilus oryzae*	XP_030760484.1	2 × 10^−^^176^	98.33

## Data Availability

Not applicable.

## Supplementary Materials

The following supporting information can be downloaded at: https://www.mdpi.com/article/10.3390/insects13030259/s1, Table S1: The primers used in this study. Table S2: The host volatiles used in the substrate competitive experiment. Table S3: The degradation abilities of recombinant SzeaGSTd1 to capryl alcohol, benzaldehyde and vanillin. Figure S1: The relative expression levels of other SzeaGSTs in different tissues. Figure S2: Expression and purification of recombinant SzeaGSTd1. Figure S3: Degradation abilities of recombinant SzeaGSTd1 for capryl alcohol. Figure S4: Degradation abilities of recombinant SzeaGSTd1 for benzaldehyde and vanillin.
