# Supplementary material for: Identification and Functional Analysis of Glutathione S-Transferases from Sitophilus zeamais in Olfactory Organ
Source: Insects. 2022 Mar 5;13(3):259. doi: 10.3390/insects13030259 (PMC8950995; doi:10.3390/insects13030259)
Supplement: Supplementary file 1 [file insects-13-00259-s001.zip › insects-1447199-supplementary.pdf]

Supporting Information

Table S1. The primers used in this study.

Primer name	Primer sequences (5′→3′)	Primer used
SzeaGSTd1-qF	GCGCGTGCTATCTATTTCCG	For RT-qPCR
SzeaGSTd1-qR	CGGCTACAGTTAGTGTGGGG	
SzeaGSTd2-qF	TTGGGTCGCTAGATGTGCTT	
SzeaGSTd2-qR	GCTGTCCAGGGGCTAACTTG	
SzeaGSTd3-qF	GATCAACCCTCAGCACACCA	
SzeaGSTd3-qR	CTTGCCGTAGGCGTTGACTA	
SzeaGSTe1-qF	TATTTCGAGGCTGCTCCACC	
SzeaGSTe1-qR	TGCCAATTGTTCCTTCTCCGA	
SzeaGSTe2-qF	ACTTGATGGCCGGTGAACAT	
SzeaGSTe2-qR	TATGCGTTGATCGCGTGACT	
SzeaGSTe3-qF	TGGTACACAAATACGGCCCC	
SzeaGSTe3-qR	AACGCTGTTCACTCTAGGGC	
SzeaGSTe4-qF	TATCACGGGCAAAAGGGACC	
SzeaGSTe4-qR	CCAAAGTCACTGCATCCCCT	
SzeaGSTe5-qF	TTTTATGGGACAGCCACGCT	
SzeaGSTe5-qR	ACCGTCTGAATAGCGTTCCA	
SzeaGSTs1-qF	ATCATGGGGCTAGGAGAACCT	
SzeaGSTs1-qR	CGTTGACTTCCAGAACGGGT	
SzeaGSTs2-qF	TTTCGATACACCTGGCCGTG	
SzeaGSTs2-qR	CTGGAACCTGTCCATAGGGTG	
SzeaGSTs3-qF	GGAGGATACACTGTCGGATCAC	
SzeaGSTs3-qR	CCGGGATGTCTGTTCTCCAG	
SzeaGSTz1-qF	CCTACTGGAGGAGTTCGTGC	
SzeaGSTz1-qR	CCGCCAGCTTTGATAAGGGA	
SzeaGSTt1-qF	ACTGGTAACGGCAACGATGT	
SzeaGSTt1-qR	GCGCTGATTTCCTTCATGCC	
β-actin-qF	GGGGCGAATACTGTGAGAAA	
β-actin-qR	AGCAGGTTCAAAAGGCTCAA	
SzeaGSTd1-F	CG***GGATCC***ATGTCGGGAAGCAACGAA	For prokaryotic expression
SzeaGSTd1-R	CCC***AAGCTT***TTTAATACTTGATAATTTA	

The restriction enzyme sites are in italicized bold.

Table S2. The host volatiles used in substrate competitive experiment.

Host volatiles	CAS	MW	Formula	Purity (%)	Source
1-Hexanol	111-27-3	102.17	$C_6H_{14}O$	>98 (GR)	Adamas-beta
Capryl alcohol	111-87-5	130.23	$C_8H_{18}O$	>99 (GR)	Adamas-beta
1-Hexadecanol	36653-82-4	242.44	$C_{16}H_{34}O$	>98 (GR)	Adamas-beta
Valeraldehyde	110-62-3	86.13	$C_5H_{10}O$	>98 (GR)	Adamas-beta
Heptaldehyde	111-71-7	114.19	$C_7H_{14}O$	>97 (GR)	Adamas-beta
Benzaldehyde	100-52-7	106.12	C_7H_6O	>99 (GR)	Adamas-beta
Vanillin	121-33-5	152.15	$C_8H_8O_3$	>99 (GR)	Adamas-beta
1-Nonanal	124-19-6	142.24	$C_9H_{18}O$	>95 (GR)	TCI
Decanal	112-31-2	156.27	$C_{10}H_{20}O$	>97 (GR)	Adamas-beta
Myrcene	123-35-3	136.23	$C_{10}H_{16}$	>90 (GR)	Adamas-beta

Table S3. The degradation abilities of recombinant SzeaGSTd1 to capryl alcohol, benzaldehyde and vanillin.

Host volatiles	Treat	The peak area (AU × min)	Depletion (%)
Capryl alcohol	Control	2060.78 ± 7.29	26.45 ± 4.58
	SzeaGSTd1	1515.64 ± 18.97	
Benzaldehyde	Control	321.05 ± 11.81	2.73 ± 8.01
	SzeaGSTd1	312.27 ± 15.34	
Vanillin	Control	923.21 ± 4.99	0.57 ± 2.06
	SzeaGSTd1	917.99 ± 13.58	

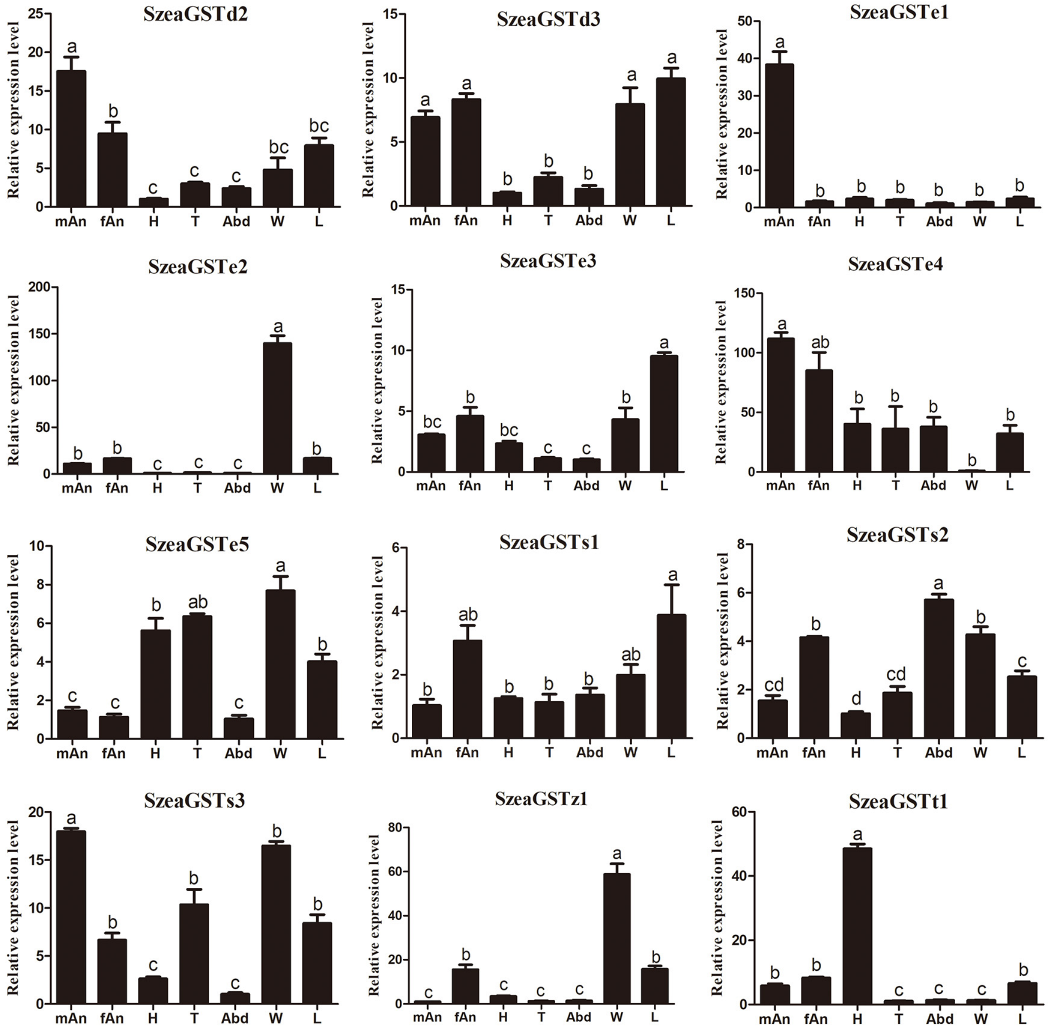

Figure S1. The relative expression levels of other *SzeaGSTs* in different tissues.

mAn: male antenna, fAn: female antenna, H: head, T: thorax, Abd: abdomen, W: wing, L: leg. The error bars represent the standard errors calculated from three replicates. Different letters on the error bars indicate significant differences analyzed by the ANOVA and HSD test ($P < 0.05$).

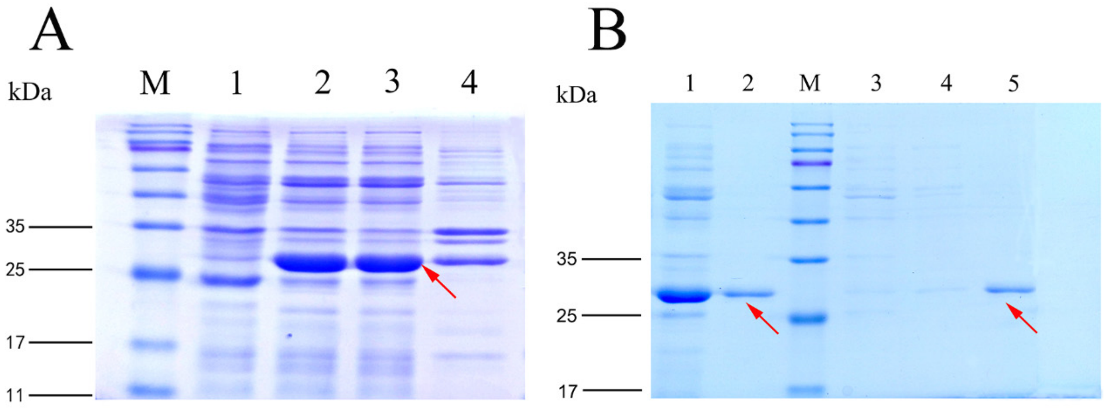

Figure S2. Expression and purification of recombinant SzeaGSTd1.

All proteins were expressed in *E. coli* BL21 using the vector pCold I and purified by Ni-His resin. A: The expression of recombinant SzeaGSTd1 protein. M: Protein weight marker, Lane 1: The whole cell lysate of pCold I-SzeaGSTd1 recombinant protein without induced, Lane 2: The whole cell lysate of pCold I-SzeaGSTd1 recombinant protein after sonication, Lane 3: Supernatant of pCold I-SzeaGSTd1 recombinant protein after induced, Lane 4: Precipitate after induced. B: Purification and SDS-PAGE analysis of recombinant protein SzeaGSTd1. M: Protein weight marker, Lane 1: Supernatant after induced, Lane 2: The purified protein of SzeaGSTd1, Lane 3: Flow through fraction, Lane 4: 100 mM imidazole elution, Lane: 200 mM imidazole elution.

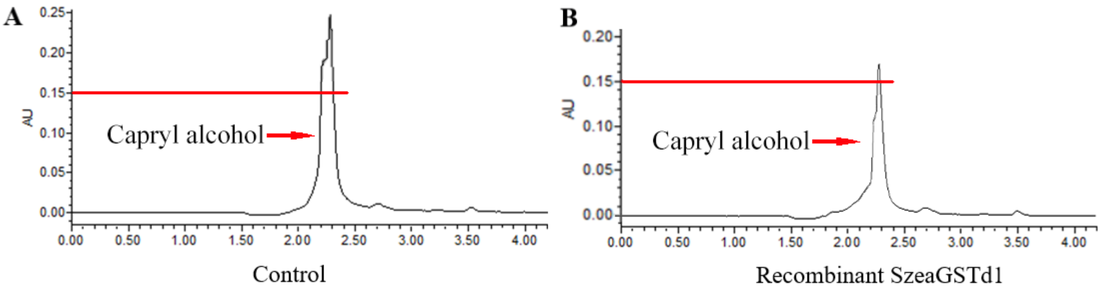

Figure S3. Degradation abilities of recombinant SzeaGSTd1 to capryl alcohol.

A: The incubation of capryl alcohol and GSH with 100 mmol/L PBS buffer. B: The incubation of capryl alcohol and GSH with recombinant SzeaGSTd1.

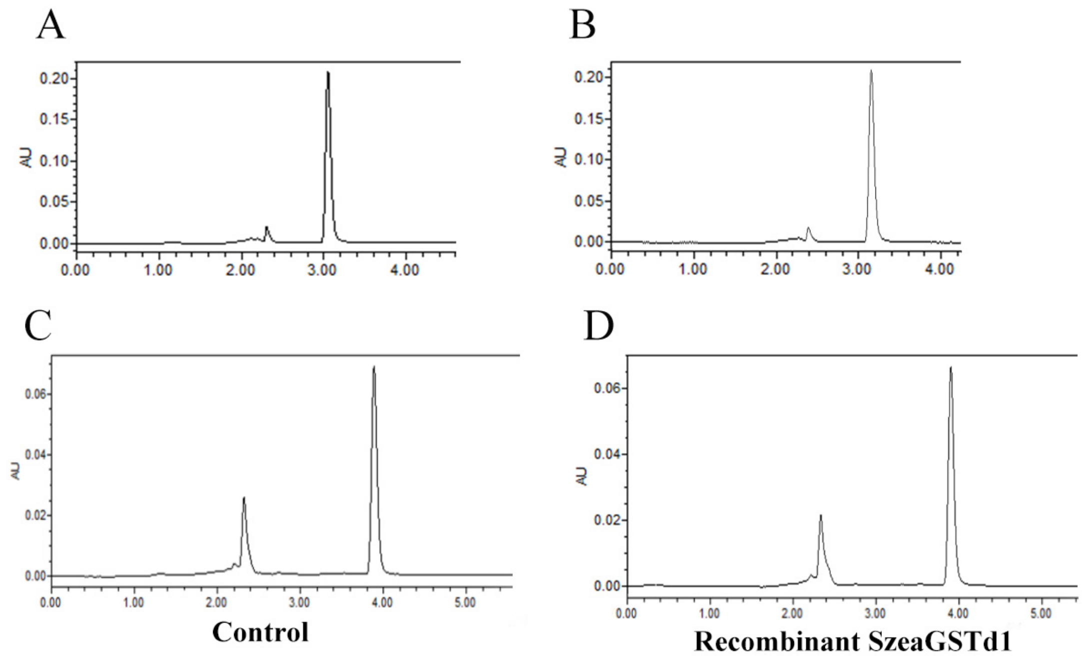

Figure S4. Degradation abilities of recombinant SzeaGSTd1 to benzaldehyde and vanillin.

A and B: Benzaldehyde. C and D: Vanillin
